# Performance of Multiparametric Functional Imaging and Texture Analysis in Predicting Synchronous Metastatic Disease in Pancreatic Ductal Adenocarcinoma Patients by Hybrid PET/MR: Initial Experience

**DOI:** 10.3389/fonc.2020.00198

**Published:** 2020-02-25

**Authors:** Jing Gao, Xinyun Huang, Hongping Meng, Miao Zhang, Xiaozhe Zhang, Xiaozhu Lin, Biao Li

**Affiliations:** Ruijin Hospital, Shanghai Jiao Tong University School of Medicine, Shanghai, China

**Keywords:** pancreas—adenocarcinoma, metastasis, PET/MR hybrid imaging, multiparametric, texture analysis

## Abstract

**Objectives:** To assess the imaging biomarkers of glucose metabolic activity and diffusion-weighted imaging (DWI) derived from pretreatment integrated ^18^F-fluorodeoxyglucose positron emission tomography-magnetic resonance (^18^F-FDG PET/MR) imaging as potential predictive factors of metastasis in patients with pancreatic ductal adenocarcinoma (PDAC).

**Patients and Methods:** We retrospectively included 17 consecutive patients with pathologically confirmed PDAC by pretreatment ^18^F-FDG PET/MR. The study subjects were divided into a non-metastatic group (M0, six cases) and a metastatic group (M1, 11 cases). The ^18^F-FDG PET/MR images were reviewed independently by two board certificated nuclear medicine physicians and one radiologist. Conventional characteristics and quantitative parameters from both PET and apparent diffusion coefficient (ADC) were assessed. The texture features were extracted from LIFEx packages (www.lifexsoft.org), and a 3D tumor volume of interest was manually drawn on fused PET/ADC images. Chi-square tests, independent-samples *t*-tests and Mann–Whitney *U*-tests were used to compare the differences in single parameters between the two groups. A logistic regression analysis was performed to determine independent predictors. A receiver operating characteristic (ROC) curve analysis was performed to assess the discriminatory power of the selected parameters. Correlations between metabolic parameters and ADC features were calculated with Spearman's rank correlation coefficient test.

**Results:** For conventional parameters, univariable analysis demonstrated that the M1 group had a significantly larger size and a higher peak of standardized uptake value (SUV_peak_), metabolic tumor volume (MTV), and total lesion glycolysis (TLG) than those of the M0 group (*p* < 0.05 for all). TLG remained significant predictor in the multivariable analysis, but there were no significant differences for the area under the ROC curve (AUC) among the four conventional features in differential diagnoses (*p* > 0.05 for all). For the texture features, there were four features from the PET image and 13 from the ADC map that showed significant differences between the two groups. Multivariate analysis indicated that one feature from PET and three from the ADC were significant predictors. TLG was associated with ADC-GLRLM_GLNU (*r* = 0.659), ADC-GLRLM_LRHGE (*r* = 0.762), and PET-GLRLM_LRHGE (*r* = 0.806).

**Conclusions:** Multiple parameters and texture features of primary tumors from ^18^F-FDG PET/MR images maybe reliable biomarkers to predict synchronous metastatic disease for the pretreatment PDAC.

## Introduction

Pancreatic ductal adenocarcinoma (PDAC) has poor prognosis, and ranks the fourth among cancer-related death. It often presents at a late stage, and exhibits a 5-year overall survival rate of <8% (1). Distant metastasis is still frequently encountered in the operation of patients with potentially resectable PDAC (2, 3). Currently surgical resection is the only curative treatment for PADC. But it is very challenging to identify occult metastatic disease (OMD) by conventional images in the patients with resectable tumor before surgery, which makes further development of preoperative imaging essential. The accurate diagnosis of pancreatic cancer is important for determining the optimal management strategy. The predicting of patients with poor prognosis in advance would help in initial management, including the use of neoadjuvant chemotherapy or radiation, or adopting adjuvant therapy after surgery. Although OMD in PDAC is common, the mechanism and risk factors of its development are largely unknown.

Positron emission tomography/magnetic resonance (PET/MR) imaging is a newly developed technology that combines the anatomical and functional characteristics of MR imaging with the metabolic information of PET in one-stop examination. Hybrid PET/MR has been introduced into the clinical application setting since 2011. Studies on the feasibility and potential applications of PET/MR imaging have been reported soon after that, and oncology was one of the hot topic (4–8). Because multiparametric PET/MR imaging can provide many biomarkers of the studied diseases non-invasively, it was widely used in oncological research, especially for the tumor diagnosis, treatment planning, surveillance, and follow-up. Compared with PET/CT plus contrast-enhanced multidetector CT (MDCT), ^18^F-FDG PET/MR imaging obtained a similar diagnostic performance in the preoperative staging and resectability assessment of pancreatic neoplasms (9).

MRI with diffusion weighted imaging (DWI) has incremental value in detecting small hepatic metastasis and peritoneal implants when combined with FDG PET imaging, which can avoid unnecessary surgery (10, 11). With the integration of the advantages of PET and MR imaging, PET/MR imaging bears great potential in detecting and diagnosing of metastatic disease in PDAC patients.

By extracting and analyzing a large number of putative imaging features, which may reflect the heterogeneity of tissues, texture analysis and radiomics played an increasingly important role in cancer research (12). The rationale is that image texture features and radiomics characteristics may contain information of tumor phenotypes, which can reflect patient prognosis indirectly. Texture analysis and radiomics using CT images, which are widely available, has been used to predict aggressiveness, disease-free survival (DFS), and overall survival (OS) in patients with PDAC (13–15). DWI can reflect the tissue cellularity, and has been used in texture analysis in many other studies (16–19). Quantitative parameters obtained from current-generation hybrid imaging can provide complementary information of morphology and function simultaneously, which might be related to tumor biological behavior (16, 20). In the present study, we first explored the value of three-dimensional texture analysis based on hybrid ^18^F-FDG PET/ADC images in predicting of metastatic disease in PDAC patients.

Our hypothesis is that different kind of imaging parameters and features from pretreatment multiparametric PET/MR can be used to predict synchronous distant metastasis in patients with PDAC. In addition, the automated analysis of quantitative imaging features may complement conventional imaging metrics for prognostic evaluation. The purpose of this study was to assess conventional PET/MR findings and tumor texture features on pretreatment PET/MR imaging as potential predictive factors of metastasis for PDAC.

## Materials and Methods

### Subjects

This retrospective study was approved by the Institutional Ethics Committee of Ruijin Hospital, and informed consent was obtained from the patients who participated in another clinical study (application of abdominal PET/MR sequentially after whole body ^18^F-FDG PET/CT). No written informed consent was required for the other patients who underwent whole body PET/MR according to clinical indications. From March 2018 to January 2020, 29 consecutive patients (mean age, 60.8 ± 10.1 years; men/women, 12/17) with suspected pancreatic cancer underwent hybrid multiparametric ^18^F-FDG PET/MR with DWI before treatment. The patients were considered eligible based on the following criteria: (1) histopathological examination via either biopsy or surgical procedure; (2) hybrid ^18^F-FDG PET/MR scans (with DWI) performed before biopsy and surgical intervention; and (3) no local or systemic treatments to pancreatic cancer. Of the 29 patients, 12 patients without a pathological-confirmed diagnosis were excluded. Finally, 17 patients (mean age, 57.4 ± 10.1 years; range, 40–75 years; eight men, nine women) with PDAC were included in our study population. All patients tolerated this examination. Tumor size was measured according to MRI images, and the maximum diameter was recorded. Synchronous distant metastases were confirmed with imaging techniques and, if possible, by either surgical operation or biopsy. The study subjects were divided into two groups [without synchronous distant metastasis (M0 group) and with synchronous distant metastasis (M1 group)]. The patient characteristics are summarized in Table 1.

**Table 1:** Basic characteristics of the study participants (17 cases).

**Patient number**	**Gender**	**Age (years)**	**Height (cm)**	**Body weight (Kg)**	**Tumor location**	**Tumor size (cm)**	**Location of metastasis**	**Group**
1	Female	62	164	55	Body/tail	4.7	Peritoneum	Metastatic
2	Female	40	154	40	Body/tail	5.1	Liver	Metastatic
3	Male	66	170	65	Head/neck	4.3		Non-metastatic
4	Male	61	172	57	head/neck	4.6		Non-metastatic
5	Male	66	170	57	Head/neck	3.9	Liver	Metastatic
6	Female	55	160	45	Body/tail	5.7	peritoneum	metastatic
7	Female	47	159	60	Head/neck	2.2		Non-metastatic
8	Male	49	173	64	Body/tail	4.6	Multiple*	Metastatic
9	Male	72	170	78	Head/neck	2.5		Non-metastatic
10	Female	75	160	55	Body/tail	4.5	Supraclavicular lymph node	Metastatic
11	Female	43	155	56	Head/neck	3.2		Non-metastatic
12	Male	57	170	66	Body/tail	6.0	Peritoneum	Metastatic
13	Female	51	164	47	Body/tail	4.7	Liver	Metastatic
14	Male	63	170	70	Body/tail	4.0	Liver	Metastatic
15	Female	65	163	60	Body/tail	2.7	Liver	Metastatic
16	Male	48	180	69	Head/neck	3.3		Non-metastatic
17	Female	56	160	47	Body/tail	3.2	Liver, peritoneum	Metastatic

Multiple*, liver, left adrenal gland, remote lymph nodes, bones.

### PET/MR Protocol

Whole-body PET/MR was performed using an integrated PET/MR system (Biograph mMR; Siemens Healthineers, Erlangen, Germany). All participants were fasted for at least 6 h before the study and given intravenous ^18^F-FDG 2.5 to 6 MBq/kg at 40–100 min before each PET/MR study. For whole body examination, PET was performed from the mid-thighs to the skull base in four bed positions (acquisition time, 4 min/position) with the patient in a supine arm-down position, and head was scanned with 1 bed position for 8 min. Simultaneous MRI with axial T2-weighted 2D half-Fourier acquisition single-shot turbo spin-echo sequences(HASTE), axial DWI with echo planar sequence(b-values, 50 and 800 s/mm^2^), and axial T1-weighted imaging (T1WI) with a DIXON sequence were performed and PET data were acquired at each bed position. For abdominal examination, the simultaneous acquisition of PET and MRI data was performed. Unenhanced studies, including coronal T2WI half-Fourier acquisition single-shot fast spin-echo, axial and coronal T2WI with fat saturation, axial T1-weighted fat-suppressed three-dimensional gradient-recalled echo imaging were performed. DWI was performed by using a single-shot echo-planar imaging sequence with *b* values of 50 and 800 sec/mm^2^. The ADC map was calculated using a monoexponential function (b-values, 50 and 800 s/mm^2^; Supplementary Table 1).

The PET images were reconstructed with an ordered-subset, expectation-maximization, iterative algorithm (4 iterations, 21 subsets), with a 4-mm post reconstruction Gaussian filter and a matrix of 172 ^*^ 172. Attenuation correction of PET data was obtained by a 4-tissue-class (air, lung, fat, soft tissue) segmented attenuation map from a 2-point Dixon MR pulse sequence. Eight patients were subjected to abdominal PET/MR (after whole body PET/CT), one patient was subjected to whole body PET/MR, and eight patients were subjected to whole body plus abdominal PET/MR.

### Image Analysis

The focal ^18^F-FDG uptake at the primary tumor, the lymph nodes and distant metastases were reviewed independently by two board certificated nuclear medicine physicians (12 and 4 years of experience) on PET/MR images. A radiologist who specialized in abdominal MRI with 13 years of experience and 2 years of experience in nuclear medicine read the PET/MR studies. The nuclear medicine physicians and radiologists independently performed their analyses on the workstation. Any disagreement was resolved by discussion. The volume of interest (VOI) was manually drawn on the PET image, and a region of interest (ROI) was drawn manually on ADC maps with consensus by three readers, and the ADC values and PET parameters of the pancreatic tumor were measured.

The PET-related parameters included maximum standardized uptake value (SUV_max_), mean SUV (SUV_mean_), maximum average SUV within a 1 cm^3^ spherical volume (SUV_peak_), standard deviation of SUV(SUV_sd_), MTV, and TLG. The SUV_max_ and SUV_mean_ were defined as the maximum and mean radioactivity concentration of images enclosed by the VOI divided by the whole body concentration of the injected radioactivity. SUV_max_, SUV_mean_, SUV_peak_, and MTV values were then measured automatically using commercial software (Syngo Via Workstation; Siemens Healthineers, Erlangen, Germany). The peak of the SUV (SUV_peak_) was determined using a 1 cm^3^ spherical volume of interest automatically centered on the tumor area with the maximum uptake. The MTV was determined by segmentation of the tumor based on a 40% threshold of SUV_max_. TLG was calculated as SUV_mean_ ∗ MTV.

To measure the ADC, ROIs were manually drawn on the ADC map along the contour of the tumor on a single slice containing the largest area of the tumor. The DWI parameters included the mean ADC (ADC_mean_), standard deviation of ADC value (ADC_sd_), and minimum ADC (ADC_min_). The lowest ADC value in an ROI, ADC_min_, represented the greatest tumor cellularity.

Among all 17 patients enrolled, the following imaging biomarkers were recorded for the primary tumor: SUV_mean_, SUV_max_, SUV_peak_, SUV_sd_, MTV, TLG, ADC_mean_, ADC_min_, ADC_sd_, and tumor size (maximum diameter of the tumor from MRI). A total of 10 PET/MR parameters were applied for differentiation.

TNM staging system of American Joint Committee on Cancer (8th edition) was applied for the study patients by a multidisciplinary team for pancreatic cancer at our hospital. Among those who did not receive curative surgery, the stage was determined by biopsy and all available image results.

### Computerized Textual Analysis

Features of the primary tumor were extracted using the Local Image Features Extraction (LIFEx) package (http://www.lifexsoft.org). The texture analysis was performed inside the VOI retrieved from the fused PET/ADC images. The VOI was manually drawn with consensus by three nuclear medicine-certified physicians and radiologist together. Histogram-based features, the gray-level cooccurrence matrix (GLCM), the neighborhood gray-level different matrix (NGLDM), the gray level run length matrix (GLRLM) and the gray level zone length matrix (GLZLM) were obtained. There were 37 texture indices analyzed in this study (Supplementary Table 2). The^18^F-FDG uptake intensity data were rescaled using 64 discrete values to reduce the image noise.

### Statistical Analysis

Summary statistics are presented as the mean ± SD for quantitative variables or frequency for qualitative variables. Appropriate statistical tests were used to assess differences in ^18^F-FDG PET/MR imaging biomarkers between patients with and without synchronous metastatic disease. We first performed univariate analyses on a series of variables, followed by multivariate analyses on selected variables with significant differences in the univariate analysis. The patient gender and tumor location between two groups were compared using the Chi-square test with Fisher's exact test. The patient age, height, body weight, and tumor size between the two groups were compared using an independent-samples *t*-test. The ADC values, PET parameters, and textural parameters between the two groups were compared using the independent-samples Mann–Whitney *U*-test. Multivariable analysis was investigated using the stepwise forward logistic regression model with significant parameters. Receiver operating characteristic (ROC) analyses were performed to evaluate the diagnostic accuracy of predicting synchronous metastatic disease (M1 or M0), and the area under the ROC curve (AUC) was calculated to identify the optimal cut-off values for each parameter. The parameter was most likely to accurately identify a positive instance (with synchronous metastatic disease) when the AUC value was high (*p* < 0.05). The 95% confidence intervals (CI) for AUC and *p-*values for comparison of related ROC curves were obtained with the method described by DeLong and coworkers (21). The relationship between metabolic parameters and texture features from the ADC map was also evaluated using Spearman's rank correlation coefficient test. A *p* < 0.05 was considered statistically significant, and all *p-*values presented were two-sided. Data were analyzed using SPSS software (SPSS for Windows 23; IBM Corp., Armonk, USA) and MedCalc for Windows, version 11.4 (MedCalc Software, Ostend, Belgium).

## Results

### Patient Characteristics

Six patients without synchronous metastatic disease (M0) and 11 patients with synchronous metastatic disease (M1) were included in this study. The average age was 56.2 ± 11.8 years (range from 43 to 72 years) in M0 patients and 58.1 ± 9.5 years (range from 40 to 75 years) in M1 patients. There were four males and two females in the M0 patient group and four males and seven females in the M1 patient group. The age, gender, height, and body weight did not differ significantly between the two groups (*p* > 0.05 for all). The characteristics of the patients are summarized in Table 1.

### Conventional Parameters

**Figure 1:**
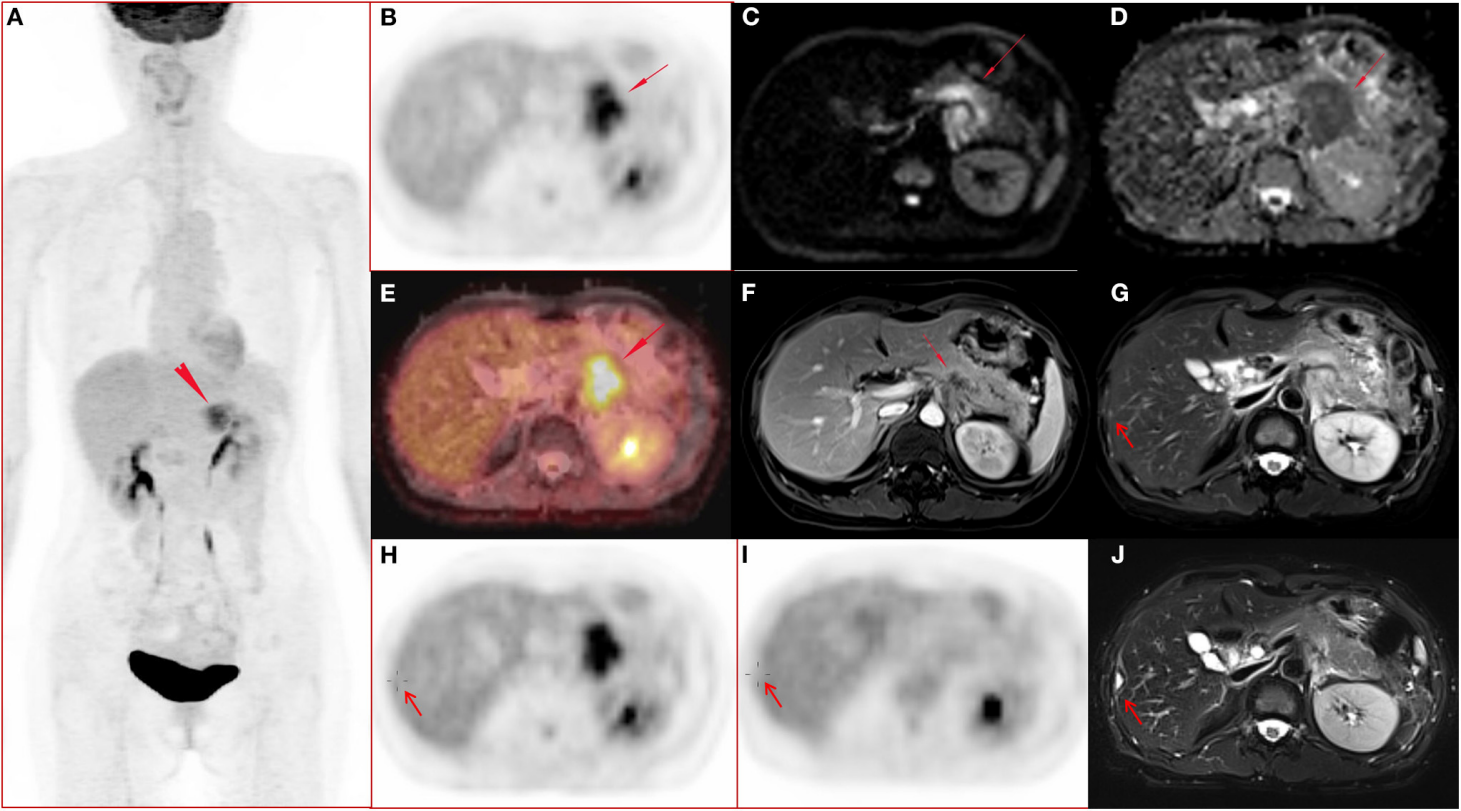
A 51-year-old female with pancreatic ductal adenocarcinoma in body and tail with hepatic metastases. **(A–J)** Whole body PET image with maximum intensity projection (MIP) **(A)** and axial abdominal PET image **(B)** showed FDG metabolism increased lesion in pancreatic body and tail, with SUV_max_ 7.52, SUV_peak_ 6.39, and MTV 17.43 cm^3^. Diffusion weighted imaging (DWI, b = 800) **(C)** and apparent diffusion coefficient (ADC) map **(D)** showed a diffusion restricted lesion in pancreatic body and tail. **(E)** Fused image of PET and ADC showed a diffusion restricted lesion with hyper FDG metabolism. **(F)** Contrast enhanced (CE) T1 weighted image (T1WI) with fat suppression (fs) on late arterial phase showed hypo-vascular lesion and dilated main pancreatic duct, and the maximum diameter of the lesion was 4.7 cm. **(G,H)** Metastasis in the right lobe of the liver (arrow) confirmed by surgery operation (2 days after the initial PET/MR examination) and histo-pathological examination, and the lesion showed slightly hyper-intensity on T2 weighted image with fat saturation **(G)**, no FDG avid lesion on PET image **(H)**. (**I–J)** Follow up PET/MR 112 days after operation showed the operated region with hyper-intensity in T2 weighted image with fat saturation **(J)** and without abnormal FDG uptake on PET image **(I)**.

Tumor location, tumor size, SUV_peak_, MTV, and TLG differed significantly (*p* < 0.05 for all) between M0 and M1 patients. More tumors were located in the body/tail in the M1 group than in the M0 group (*p* = 0.001). The M1 group showed a larger tumor size than that in the M0 group (*p* = 0.039). Patients with synchronous metastatic disease demonstrated increased SUV_peak_, MTV, and TLG in the primary tumor. SUV_max_, SUV_mean_, and SUV_sd_ did not differ significantly between the two groups (*p* > 0.05 for all). ADC_mean_, ADC_min_, and ADC_sd_ did not differ significantly between the two groups (*p* > 0.05 for all). Table 2 shows the conventional quantitative parameters of the two groups. Three of the 11 patients in M1 group had FDG-negative metastatic lesions. One patient had metastatic foci in the liver (Figure 1), and two patient had metastatic peritoneal lesions. One of the six patients in M0 group had FDG-negative primary tumors (Tables 1,2).

**Table 2:** The diagnostic performance of conventional quantitative ^18^F-FDG PET/MR parameters for predicting synchronous distant metastasis in pancreatic ductal adenocarcinoma patients.

**Parameter**	**Comparison of mean value**			**Receiver operating characteristic (ROC) analysis**					
	**M0 group**	**M1 group**	***p***	**AUC**	**95% CI**	***p***	**Optimal cutoff value**	**Se (%)**	**Sp (%)**
Age (years)	56.2 ± 11.8	58.1 ± 9.5	0.719^†^						
Height (cm)	167.7 ± 9.1	164.4 ± 5.8	0.373^†^						
Body weight (Kg)	64.2 ± 8.4	55.1 ± 9.5	0.07^†^						
Tumor size (cm)	3.4 ± 1.0	4.5 ± 1.0	**0.039**^†^	0.803	0.543–0.952	0.006	>3.3	81.8	66.7
SUV_mean_	2.6 ± 1.1	3.6 ± 1.3	0.149^‡^						
SUV_max_	4.5 ± 2.0	6.3 ± 2.3	0.216^‡^						
SUV_peak_	3.0 ± 1.1	4.8 ± 1.5	**0.037**^‡^	0.818	0.560–0.960	0.004	>4.06	72.7	100.0
SUV_sd_	0.6 ± 0.3	0.8 ± 0.3	0.149^‡^						
MTV	8.4 ± 6.1	20.7 ± 13.1	**0.037**^‡^	0.818	0.560–0.960	0.003	>15.04	63.6	100.0
TLG	21.3 ± 16.7	67.7 ± 42.1	**0.020**^‡^	0.848	0.595–0.973	<0.001	>41.3	72.7	100.0
ADC_mean_ (s/mm^2^)	1192 ± 625	1311 ± 219	0.884^‡^						
ADC_min_ (s/mm^2^)	1093 ± 274	974 ± 334	0.733^‡^						
ADC_sd_ (s/mm^2^)	128 ± 25	134 ± 20	0.525^‡^						

† Independent-samples t-test, bold value indicates p-value is significant <0.05;

‡ *Independent-samples Mann-Whitney U-test, bold value indicates p-value is significant <0.05*.

*ADC, apparent diffusion coefficient; ADC_mean_, mean apparent diffusion coefficient; ADC_min_, minimum apparent diffusion coefficient; ADC_sd_: standard deviation of apparent diffusion coefficient; AUC, area under receiver operating characteristic (ROC) curve; M0, no synchronous distant metastasis; M1, with synchronous distant metastasis; MTV, metabolic tumor volume; Se, sensitivity; Sp, specificity; SUV, standardized uptake values; SUV_max_, maximum standardized uptake value; SUV_mean_, mean standardized uptake value; SUV_peak_, the peak of SUV in 1 ml; SUV_sd_, standard deviation of standardized uptake value; TLG, total lesion glycolysis; 95% CI, 95% confidence interval*.

**Figure 2:**
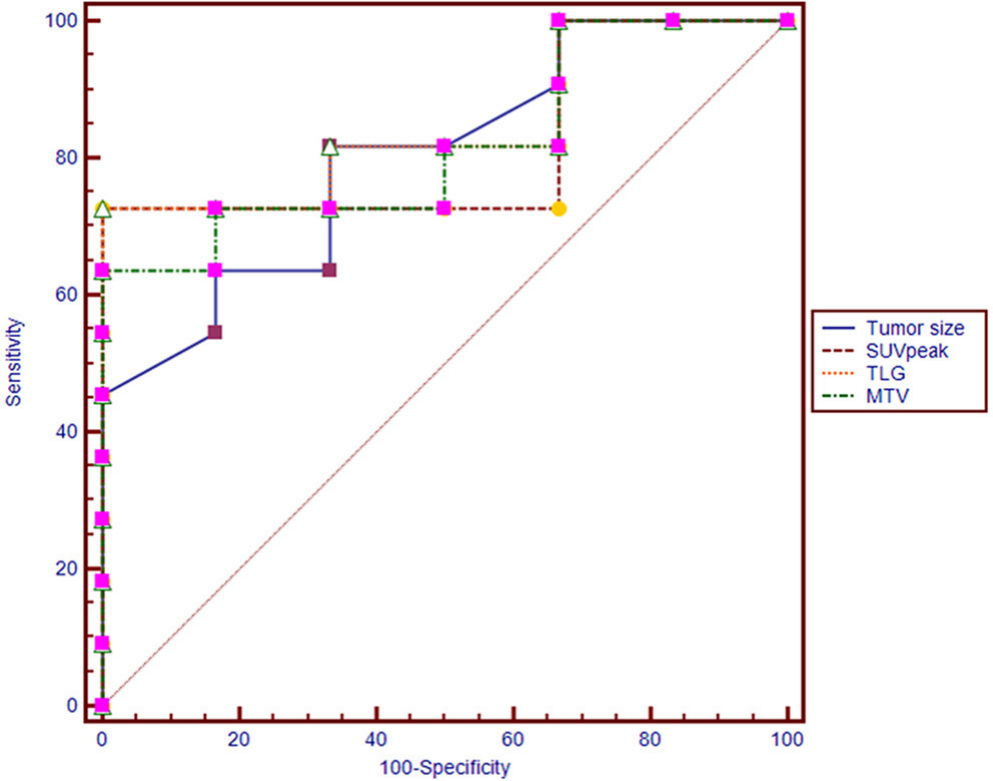
Receiver operating characteristic (ROC) curves of tumor size, SUV_peak_, MTV, and TLG for diagnosing synchronous metastatic disease in pancreatic ductal adenocarcinoma.

The conditional logistic regression model using significant parameters identified TLG as an independent predictor for synchronous metastatic disease diagnosis. The other parameters did not reach significance. Based on multivariate regression analysis, and we performed an ROC analysis for the selected parameters. The AUC was 0.848 for TLG. When the optimal cut-off point was 41.3, the TLG showed a sensitivity of 72.7% and a specificity of 100.0% (Table 2). There were no significant differences in the AUC among tumor size, SUV_peak_, MTV, and TLG (*p* > 0.05 for all; Figure 2).

### Texture Features

**Figure 3:**
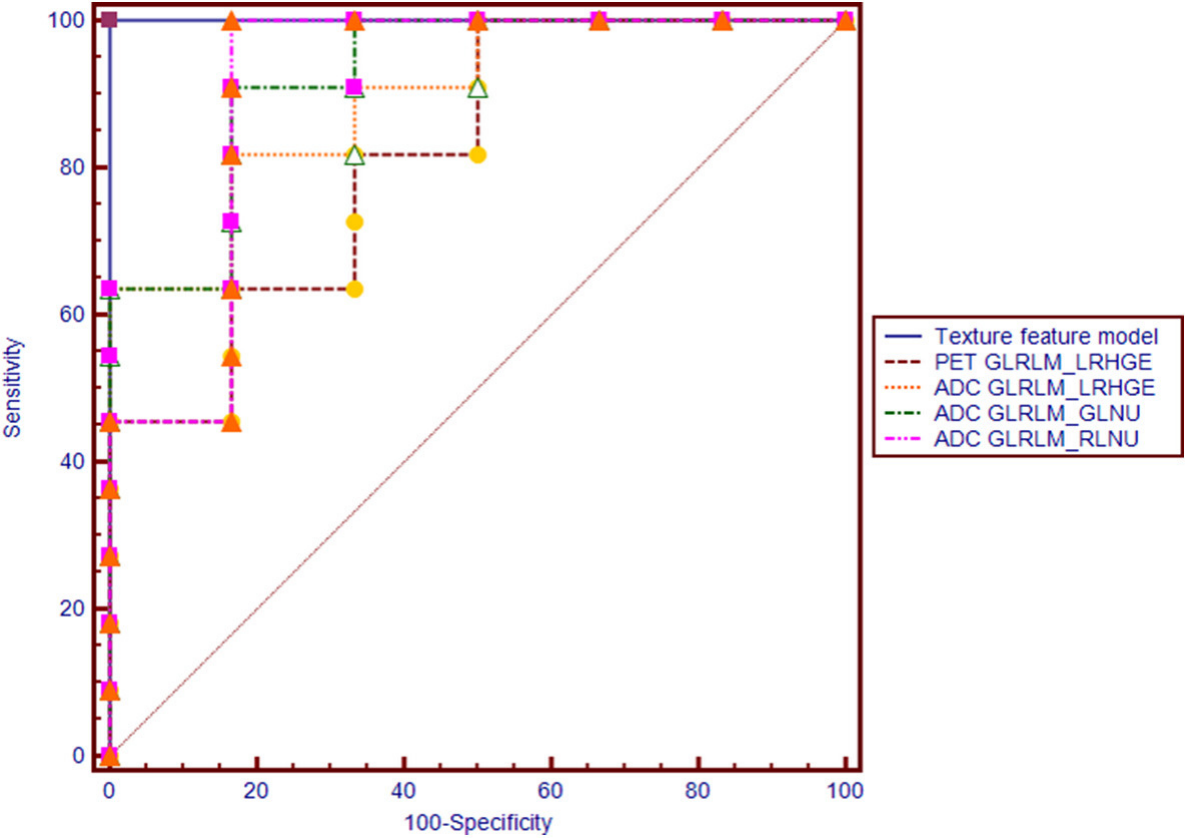
Receiver operating characteristic (ROC) curves of PET-GLRLM_LRHGE, ADC-GLRLM_LRHGE, ADC-GLRLM_GLNU, ADC-GLRLM_RLNU, and logistic regression model with combination of four texture features for diagnosing synchronous metastatic disease in pancreatic ductal adenocarcinoma.

Regarding the texture features, four features from the PET image (two GLRLM, one NGLDM and one GLZLM) and 13 features from the ADC map (two histogram based, seven GLRLM, and four GLZLM) showed significant differences between the two groups (Table 3 and Supplementary Table 3). Conditional logistic regression analysis demonstrated that Long-Run High Gray-level Emphasis of Gray-Level Run Length Matrix (GLRLM_LRHGE) from PET image, Long-Run High Gray-level Emphasis (LRHGE), Gray-level Non-Uniformity for run (GLNU), and Run Length Non-Uniformity (RLNU) of Gray-Level Run Length Matrix (GLRLM) from the ADC map were significant independent predictors for predicting synchronous metastatic disease in PDAC. The metastatic group showed significantly higher PET-GLRLM_LRHGE, ADC-GLRLM_LRHGE, ADC-GLRLM_GLNU, and ADC-GLRLM_RLNU (*p* < 0.05 for all). The AUC was 0.939, 0.894, 0.924, and 0.909 for PET-GLRLM_LRHGE, ADC-GLRLM_LRHGE, ADC-GLRLM_GLNU, and ADC-GLRLM_RLNU, respectively. The logistic regression model with proposed features obtained an AUC of 1.000 (95% CI 0.805–1.000, *p* < 0.001), but there were no significant differences in the AUC for a single parameter vs. that for the logistic regression model (*p* > 0.05 for all, Figure 3).

**Table 3:** The diagnostic performance of texture features derived from simutanous^18^F-FDG PET image and the ADC map for predicting synchronous distant metastasis in pancreatic ductal adenocarcinoma patients.

**Texture feature**	**M0 group**	**M1 group**	**P1**	**AUC**	**95% confidence intervals**	**P2**	**Optimal cut-off value**
PET-GLRLM_RLNU	467 ± 173	1391 ± 738	0.002	0.939	0.711–0.998	<0.0001	>751
PET-GLRLM_LRHGE	172 ± 77	288 ± 96	0.037	0.818	0.560–0.960	0.005	>120.6
PET-NGLDM_Coarseness	0.015 ± 0.008	0.007 ± 0.003	0.037	0.803	0.543–0.952	0.034	< =0.01
PET-GLZLM_GLNU	5.4 ± 2.3	11.3 ± 6.8	0.020	0.848	0.595–0.973	0.0004	>8.3
ADC-HISTO_Skewness	−0.02 ± 0.59	0.72 ± 0.80	0.048	0.795	0.534–0.948	0.001	>0.14
ADC-HISTO_Kurtosis	3.32 ± 1.16	5.25 ± 2.01	0.048	0.803	0.543–0.952	0.008	>3.84
ADC-GLRLM_LRE	36 ± 10	66 ± 24	0.007	0.894	0.650–0.989	<0.0001	>42.98
ADC-GLRLM_SRHGE	786 ± 51	654 ± 131	0.048	0.803	0.543–0.952	0.006	< =691
ADC-GLRLM_LRLGE	0.008 ± 0.002	0.019 ± 0.009	0.005	0.879	0.631–0.984	<0.0001	>0.012
ADC-GLRLM_LRHGE	0.15E+6 ± 0.04E+6	0.28E+6 ± 0.10E+6	0.007	0.894	0.650–0.989	<0.0001	>0.18E+6
ADC-GLRLM_GLNU	227 ± 96	442 ± 142	0.003	0.924	0.690–0.996	<0.0001	>269.4
ADC-GLRLM_RLNU	30 ± 8	45 ± 9	0.005	0.909	0.670–0.993	<0.0001	>32.6
ADC-GLRLM_RP	0.212 ± 0.023	0.165 ± 0.033	0.007	0.879	0.631–0.984	<0.0001	<0.18
ADC-GLZLM_LZE	1.53E+6 ± 1.67E+6	9.50E+6 ± 8.71E+6	0.010	0.879	0.631–0.984	<0.0001	>4.62E+6
ADC-GLZLM_LZLGE	362 ± 395	2249 ± 2061	0.010	0.879	0.631–0.984	<0.0001	>1094.1
ADC-GLZLM_LZHGE	0.65E+10 ± 0.70E+10	4.01E+10 ± 3.68E+10	0.010	0.879	0.631–0.984	<0.0001	>1.95E+10
ADC-GLZLM_ZP	0.0016 ± 0.0017	0.0006 ± 0.0004	0.050	0.795	0.534–0.948	0.010	< =0

*ADC, apparent diffusion coefficient; AUC, area under receiver operating characteristic (ROC) curve; FDG, fluorodeoxyglucose; M0, no synchronous distant metastasis; M1, with synchronous distant metastasis; NA, not applicable; P1, P-value for independent- samples Mann-Whitney U-test, indicates p-value is significant <0.05; P2, p-value for AUC, indicates P-value is significant <0.05; PET, positron emission tomography; 95% CI, 95% confidence interval; GLRLM, Gray level run length matrix; GLZLM, Gray level zone length matrix; SRHGE, Short-nun high gray-level emphasis; GLNU, Gray-level non-uniformity; RLNU, Run length non-uniformity; LZE, Long-zone emphasis; NGLDM, Neighborhood gray-level different matrix; SZHGE, Short-zone high gray-level emphasis; LZHGE, Long-zone high gray-level emphasis*.

### Correlations Between PET/MR Parameters and Texture Features

The PET parameter of TLG showed positive correlations with the texture feature of ADC-GLRLM_GLNU (*r* = 0.659, *P* = 0.004), ADC-GLRLM_LRHGE (*r* = 0.762, *P* < 0.001), and PET-GLRLM_LRHGE (*r* = 0.806, *P* < 0.001).

## Discussion

In this study, we demonstrated differences in multiparametric ^18^F-FDG PET/MR imaging biomarkers obtained from the primary tumor of PDAC between patients with and without synchronous metastasis. Then, we identified prognostic PET/MR imaging signatures in patients with PDAC by using conventional parameters and a texture analysis approach. We found that metastatic PDAC patients showed significantly larger tumor sizes, more frequent body/tail locations and higher SUV_peak_, MTV, and TLG values in the primary tumor than those in non-metastatic patients (*p* < 0.05 for all). In addition, TLG remained significant predictor in the multivariable analysis. Regarding the texture features, we found that GLRLM_RLNU, GLRLM_GLNU, and GLRLM_ LRHGE from the ADC map, and GLRLM_LRHGE from PET image were also significant predictors of synchronous metastatic disease. In addition, TLG was associated with ADC-GLRLM_GLNU, ADC-GLRLM_LRHGE, and PET-GLRLM_LRHGE.

Regarding the tumor size and location, our results were consistent with previous studies (2, 3, 22, 23). The larger the tumor, the more likely it is to have distant metastasis. The cut-off value of tumor size was similar between our study (3.3 cm) and the studies of Liu et al. (4.0 cm) and Karabicak et al. (4.2 cm) (2, 22). In a cohort of 1,423 patients with PDAC who underwent pancreatectomies, the occurrence of occult metastatic disease in PDAC accounted for 8% of cases, and multivariable analysis defined four independent predictors for occult metastatic disease (3). Patients with abdominal pain, preoperative CA 19-9 > 192 U/ml, tumor bigger than 3 cm, and indeterminate lesions on preoperative CT had high risk of occult metastatic disease (3). The cut-off value of tumor size was slightly smaller in the study of Gemenetzis et al. (3) than that in our study, which might be because that the patients were potentially resectable with occult but not obvious metastasis and the sample size was large in that study. Another study of 110 patients with PDAC (22), patients with high CA 19-9 levels and large size tumor located in body-tail are at greater risk for latent distant organ metastasis or peritoneal metastasis. Tumors located in the body/tail of the pancreas are more likely to metastasize (22, 23), which was also confirmed in our study. The metabolic parameters of ^18^F-FDG PET could reflect biological aggressiveness and predict prognosis in various studies (24–29), and we demonstrated similar results in this study. A study of 93 patients with pathologic T3 (pT3) resectable pancreatic cancer showed that tumor with high MTV2.5 is associated with both lymph node metastasis and early systemic metastasis (24). Patients who developed metastatic disease during follow-up after chemoradiotherapy had higher SUV_max_ (3.8 vs. 8.6), SUV_peak_ (2.5 vs. 7.5), SUV_mean_ (1.8 vs. 3.3), SUV_median_ (1.7 vs. 3.0), and TLG (26.9 vs. 115.9) than did those without metastatic disease (25). The average SUV_peak_ was 3.0 and 4.8 for M0 and M1 group in our study. The SUV_peak_ of PDAC without metastasis was similar between the two studies, and the SUV_peak_ of metastatic PDAC was slightly higher in the study of Wilson et al. (25) than that of the present study. Other recent studies (26–29) which made use of the PET/CT technique, unlike PET/MR, as was the case in our study, have addressed PET-derived parameters (TLG, MTV, or SUV_peak_) as independent predictors for OS and PFS outcome in patients with pancreatic adenocarcinoma. A PET/CT scoring system with combination of quantitative parameters helps to improve the prognostication significantly (28).

According to our knowledge only two studies about overall survival(OS), prognosis, and imaging biomarkers of PDAC and periampullary cancer have been published using integrated PET/MR imaging (30, 31). In a study with 60 PET/MRI of pancreatic and periampullary cancer patients, the imaging biomarkers (ADC_min_, Choline levels, TLG, MTV, MTV/ADC_min_ ratio) may predict clinical stage and progression-free survival (PFS) of the patients (30). Recently, Chen et al. have showed that multiparametric PET/MR imaging biomarkers of pancreatic cancer patients were associated with their OS (31). The application of PET/MR has just started, and more research is needed to find out the potential value of PET/MR. And more sophisticated methods are needed to improve the existing diagnostic capabilities. Radiomics in nuclear medicine is fastly developing. The advantage of radiomics should be fully explored from now on to improve the clinical value of multiparametric imaging, such as PET/CT and PET/MR, in predicting disease phenotypes and personalized diagnosis and treatment. In this study, texture analysis showed significant differences between M0 and M1 PDAC for two first-level (histogram skewness and kurtosis from ADC map) and for 15 third-level features(four from PET and 11 from ADC map). ADC-HISTO_Skewness and ADC-HISTO_Kurtosis were the first-level features with significant differences between the two groups based on the ROC analysis. According to the literature, ADC histogram analysis has the potential to provide valuable information on tumor biology and to predict tumor behavior in several malignancies (17, 18, 32, 33). The skewness and kurtosis were higher in cervical cancer patients with metastatic lymph nodes than those with negative nodal status (33). Another study showed that skewness and kurtosis of histogram analysis from ADC map were able to differentiate thyroid carcinoma with lymph node metastasis from that without metastasis (32). In the study of non-small cell lung cancer, higher ADC skewness and kurtosis were associated with lymphovascular invasion and pleural invasion (34). In a study of pediatric diffuse intrinsic potine glioma using ^18^F-FDG PET and MRI ADC histogram, higher ADC skewness and kurtosis of the enhancing portion of the tumor were associated with shorter PFS (16). HISTO_Skewness is the asymmetry of the gray-level distribution in the histogram. If the peak of the frequency distribution shifts to the left, the long tail extends to the right, which is called a positive skewed distribution. Kurtosis reflects the sharpness of the histogram peak. So in this study, in M1 patients most voxels containing an ADC less than the mean. The lower ADC value indicates the higher cellularity and aggressiveness. Unexpectedly, the conventional ADC values (ADC_mean_, ADC_min_, ADC_sd_) had no significant differences between metastatic and non-metastatic PDAC. Considering that the sample size of this pilot study is too small, it would be hasty to draw any conclusions from this negative finding.

The first-level texture feature describes the characteristics related to the voxel intensity distribution, while the meaning of second- and third-level features is non-figurative. In this study, the texture features of GLRLM_RLNU, GLRLM_GLNU, and GLRLM_LRHGE from the ADC map, and GLRLM_LRHGE from PET image were independent predictors of synchronous metastatic disease. GLRLM reflects the comprehensive information of the image grayscale with respect to direction, adjacent interval, and variation amplitude. GLRLM is a set of statistical feature extracted from medical images and applied in radiomics frequently (35–37). HGRE measures the distribution of sections of high intensity, and its value is expected to be large if the number of sections of high intensity is high. In a study of breast cancer using ^18^F-FDG PET texture analysis, triple negative breast cancer (TNBC) had higher RHGE value than non-TNBC and exhibited more tumor heterogeneity (38). Gray-level Non-Uniformity for run (GLNU) measures similarity of values of gray-level and Run Length Non-Uniformity (RLNU) measures similarity of run length. In this study, the PET-GLRLM_LRHGE, ADC-GLRLM_LRHGE, ADC-GLRLM_GLNU, and ADC-GLRLM_RLNU values were higher in M1 patients than in M0 patients. This may indicate that the higher heterogeneity in PET image and ADC map of PDAC, the higher risk of metastasize. ADC-GLRLM_LRHGE and ADC-GLRLM_GLNU were positively correlated with TLG, which indicate that PDAC with higher TLG might have higher heterogeneity of ADC map. Whether there is an intrinsic link between these texture features and tumor biological behaviors requires further research. Ultimately, like the readings of a radiologist, texture analyses should contain all image sequences. Since such research has just begun, separate and gradually deepening study may be a suitable method. Although radiomics is a promising tool for high-tech hybrid imaging technology such as PET/CT and PET/MR (39–41), but many factors such as attenuation correction techniques, different uptake times and voxel size may influence the radiomic features (42, 43), which makes the application value obscure. We need to be cautious about the results of the present study, and continue to increase the sample size and research centers to further investigate the exact value of texture features and radiomics in PET/MR.

This study has several limitations. First, this study is a retrospective study, and the number of patients is limited. Second, evaluating of serum tumor markers vs. imaging parameters was not included in this study. Third, there is a certain degree of subjectivity in manually delineating the tumor boundaries. Therefore, prospective studies with a larger sample and multicenter studies are needed to confirm the present findings. Another limitation of this study is that some of the patients underwent only abdominal PET/MR sequentially after a whole body ^18^F-FDG PET/CT.

In conclusion, our preliminary study showed that multi-parameter and textural features of primary tumors in ^18^F-FDG PET/MR images are reliable biomarkers for predicting synchronous metastatic disease in pretreatment PDAC, which might be helpful for the selection of optimal therapeutic methods. This technique may provide a convenient and non-invasive approach to evaluate the prognosis of PDAC in clinical practice. However, multicenter studies with a large population are needed to confirm these results.

## Data Availability Statement

The datasets generated for this study are available on request to the corresponding author.

## Ethics Statement

The studies involving human participants were reviewed and approved by Ruijin Hospital Ethics Committee Shanghai Jiao Tong University School of Medicine. The patients/participants provided their written informed consent to participate in this study.

## Author Contributions

XL conceived the idea of the study. XH and JG collected the data. XH, MZ, and XL performed image interpretation and analysis. JG performed the statistical analysis. JG and XL drafted the manuscript. HM and XZ scanned the patients. BL edited and reviewed the manuscript. All authors read and approved the final manuscript.

### Conflict of Interest

The authors declare that the research was conducted in the absence of any commercial or financial relationships that could be construed as a potential conflict of interest.
