# Supplementary material for: Performance of Multiparametric Functional Imaging and Texture Analysis in Predicting Synchronous Metastatic Disease in Pancreatic Ductal Adenocarcinoma Patients by Hybrid PET/MR: Initial Experience
Source: Front Oncol. 2020 Feb 25;10:198. doi: 10.3389/fonc.2020.00198 (PMC7052324; doi:10.3389/fonc.2020.00198)
Supplement: Supplementary file 1 [file Table_1.DOCX]

**Supplementary Table 1: MRI sequences and acquisition parameters.**

|  | Sequence | | | | | | | |
| --- | --- | --- | --- | --- | --- | --- | --- | --- |
|  | Whole body | | | Abdominal | | | | |
|  | Axial T2WI(HASTE) | Axial DWI-fs | Axial T1WI DOXON | Coronal T2WI (HASTE) | Coronal T2WI-TSE-fs | Axial T2WI-TSE-fs | Axial DWI-fs | Axial T1WI-fs vibe |
| Repetition time/echo time( ms) | 1400/95 | 6400/78 | 4.04/1.24 | 2000/91  1400/96 | 3912~4854/102 | 3462.5~5239.0/86 | 5310/73,  8300/72 | 4.56/2.03 |
| Flip angle | 90° | 90° | 14° | 134°~154° | 80° | 62°~86° | 90° | 9° |
| Acquisition type, scanning sequence | 2D-SE | 2D-EP | 3D-GR | 2D-SE | 2D-SE | 2D-SE | 2D-EP | 3D-GR |
| In-plane resolution(mm) | 1.25*1.25 | 1.56*1.56 | 1.24*1.24 | 1.19*1.19~  1.48*1.48 | 1.19*1.19 | 1.25*1.25 | 2.90*2.90 | 1.19*1.19 |
| Number of section | 132 | 132 | 120 | 35~25 | 25 | 30 | 28 | 72 |
| Section thickness(mm) | 6 | 6 | 6 | 5 | 3.5 | 5.5 | 5, 6 | 3 |
| b-Value(s/mm^2^) | - | 50, 800 | - | - | - | - | 50, 800 | - |
| Number of averages | 1 | 2 | 1 | 1 | 1 | 1 | 4, 3 | 1 |

*T2WI, T2 weighted imaging; HASTE, half-Fourier acquisition single-shot fast spin-echo; DWI, diffusion weighted imaging, fs, fat saturation or fat suppression; TSE, turbo spin-echo; 2D, two-dimensional;* *SE, spin-echo; 3D, three-dimensional;* *GR, gradient-recalled; EP, echo-planar; vibe, volumetric interpolated breath-hold examination*
