# Supplementary material for: Performance of Multiparametric Functional Imaging and Texture Analysis in Predicting Synchronous Metastatic Disease in Pancreatic Ductal Adenocarcinoma Patients by Hybrid PET/MR: Initial Experience
Source: Front Oncol. 2020 Feb 25;10:198. doi: 10.3389/fonc.2020.00198 (PMC7052324; doi:10.3389/fonc.2020.00198)
Supplement: Supplementary file 2 [file Table_2.DOCX]

**Supplementary Table 2: Texture features analyzed for pancreatic ductal adenocarcinoma with the Local Image Features Extraction (LIFEx) package (http://www.lifexsoft.org)**

| Classification of matrix | Index |
| --- | --- |
| Histogram-based | Skewness |
|  | Kurtosis |
|  | Entropy_log10 |
|  | Entropy_log2 |
|  | Energy |
| Gray-Level Cooccurrence Matrix (GLCM) | Homogeneity |
|  | Energy |
|  | Contrast |
|  | Correlation |
|  | Entropy_log10 |
|  | Entropy_log2 |
|  | Dissimilarity |
| Gray-Level Run Length Matrix (GLRLM) | Short-Run Emphasis (SRE) |
|  | Long-Run Emphasis (LRE) |
|  | Low Gray-level Run Emphasis (LGRE) |
|  | High Gray-level Run Emphasis (HGRE) |
|  | Short-Run Low Gray-level Emphasis (SRLGE) |
|  | Short-Run High Gray-level Emphasis (SRHGE) |
|  | Long-Run Low Gray-level Emphasis (LRLGE) |
|  | Long-Run High Gray-level Emphasis (LRHGE) |
|  | Gray-level Non-Uniformity for run (GLNU) |
|  | Run Length Non-Uniformity (RLNU) |
|  | Run Percentage (RP) |
| Neighborhood Gray-Level Different Matrix (NGLDM) | Coarseness |
|  | Contrast |
|  | Busyness |
| Gray-Level Zone Length Matrix (GLZLM) | Short-Zone Emphasis (SZE) |
|  | Long-Zone Emphasis (LZE) |
|  | Low Gray-level Zone Emphasis (LGZE) |
|  | High Gray-level Zone Emphasis (HGZE) |
|  | Short Zone Low Gray-level Emphasis (SZLGE) |
|  | Short Zone High Gray-level Emphasis (SZHGE) |
|  | Long Zone Low Gray-level Emphasis (LZLGE) |
|  | Long Zone High Gray-level Emphasis (LZHGE) |
|  | Gray-level Non-Uniformity for zone (GLNU) |
|  | Zone Length Non-Uniformity (ZLNU) |
|  | Zone Percentage (ZP) |
