# Supplementary material for: Performance of Multiparametric Functional Imaging and Texture Analysis in Predicting Synchronous Metastatic Disease in Pancreatic Ductal Adenocarcinoma Patients by Hybrid PET/MR: Initial Experience
Source: Front Oncol. 2020 Feb 25;10:198. doi: 10.3389/fonc.2020.00198 (PMC7052324; doi:10.3389/fonc.2020.00198)
Supplement: Supplementary file 3 [file Table_3.docx]

**Supplementary Table 3: Comparison of texture features from ^18^F-FDG PET image and ADC map in pancreatic ductal adenocarcinoma patients with and without synchronous metastatic disease.**

| **Parameters** | **PET**  **(Mean±standard deviation)** | | ***p*-value**  **(Independent samples Mann-Whitney U-test)** | **ADC**  **(Mean±standard deviation)** | | ***p*-value**  **(Independent samples Mann-Whitney U-test)** |
| --- | --- | --- | --- | --- | --- | --- |
|  | **M0** | **M1** |  | **M0** | **M1** |  |
| **HISTO_ Skewness** | 0.59±0.70 | 0.38±0.60 | 0.733 | -0.02±0.59 | 0.72±0.80 | **0.048** |
| **HISTO_Kurtosis** | 3.43±1.30 | 3.49±0.94 | 1.000 | 3.32±1.16 | 5.25±2.01 | **0.048** |
| **HISTO_Entropy_log10** | 0.94±0.29 | 1.02±0.17 | 0.462 | 0.002±0.004 | 0.002±0.0.004 | 1.000 |
| **HISTO_Entropy_log2** | 3.12±0.95 | 3.39±0.57 | 0.462 | 0.005±0.012 | 0.005±0.010 | 1.000 |
| **HISTO_Energy** | 0.17±0.13 | 0.12±0.06 | 0.462 | 0.998±0.004 | 0.999±0.003 | 0.808 |
| **GLCM_Homogeneity** | 0.59±0.15 | 0.57±0.10 | 0.733 | 0.999±0.002 | 0.999±0.002 | 1.000 |
| **GLCM_Energy** | 0.06±0.08 | 0.03±0.03 | 0.462 | 0.998±0.004 | 0.999±0.004 | 1.000 |
| **GLCM_Contrast** | 5.22±6.42 | 3.51±2.27 | 1.000 | 0.59±1.45 | 3.00±6.88 | 0.884 |
| **GLCM_Correlation** | 0.75±0.10 | 0.80±0.05 | 0.301 | NA | NA | NA |
| **GLCM_Entropy_log10** | 1.64±0.51 | 1.77±0.33 | 0.591 | 0.003±0.008 | 0.003±0.006 | 1.000 |
| **GLCM_Entropy_log2** | 5.44±1.69 | 5.89±1.08 | 0.591 | 0.010±0.024 | 0.008±0.018 | 0.961 |
| **GLCM_Dissimilarity** | 1.42±1.00 | 1.32±0.51 | 1.000 | 0.019±0.046 | 0.047±0.108 | 0884 |
| **GLRLM_SRE** | 0.79±0.13 | 0.82±0.08 | 0.660 | 0.187±0.014 | 0.157±0.034 | 0.078 |
| **GLRLM_LRE** | 3.15±2.51 | 2.56±1.00 | 0.961 | 36±10 | 66±24 | **0.007** |
| **GLRLM_LGRE** | 0.037±0.044 | 0.014±0.008 | 0.078 | 3.33E-3±7.57E-3 | 1.06E-3±2.72E-3 | 0.591 |
| **GLRLM_HGRE** | 89±64 | 129±64 | 0.149 | 4219±15 | 4214±25 | 0.884 |
| **GLRLM_SRLGE** | 0.025±0.023 | 0.011±0.005 | 0.078 | 2.93E-3±7.06E-3 | 0.81E-3±2.56E-3 | 0.591 |
| **GLRLM_SRHGE** | 78±62 | 108±61 | 0.301 | 786±51 | 654±131 | **0.048** |
| **GLRLM_LRLGE** | 0.21±0.40 | 0.04±0.03 | 0.098 | 0.008±0.002 | 0.019±0.009 | **0.005** |
| **GLRLM_LRHGE** | 172±77 | 288±96 | **0.037** | 0.15E+6±0.04E+6 | 0.28E+6±0.10E+6 | **0.019** |
| **GLRLM_GLNU** | 131±131 | 228±117 | 0.062 | 227±96 | 442±142 | **0.003** |
| **GLRLM_RLNU** | 467±173 | 1391±738 | **0.002** | 30±8 | 45±9 | **0.005** |
| **GLRLM_RP** | 0.74±0.14 | 0.76±0.09 | 0.808 | 0.212±0.023 | 0.165±0.033 | **0.007** |
| **NGLDM_Coarseness** | 0.015±0.008 | 0.007±0.003 | 0.037 | 0.0042±0.0102 | 0.0006±0.0016 | 0.961 |
| **NGLDM_Contrast** | 0.049±0.033 | 0.038±0.016 | 0.733 | 25.5E-5±46.2E-5 | 3.2 E-5±10.5 E-5 | 0.808 |
| **NGLDM_Busyness** | 2.42±4.21 | 1.51±1.29 | 0.350 | 0.50±1.22 | 14.90±36.31 | 0.884 |
| **GLZLM_SZE** | 0.33±0.16 | 0.34±0.10 | 1.000 | 0.11±0.27 | 0.01±0.02 | 0.961 |
| **GLZLM_LZE** | 17345±36829 | 12233±15704 | 0.404 | 1.53E+6±1.67E+6 | 9.50E+6±8.71E+6 | **0.010** |
| **GLZLM_LGZE** | 0.046±0.054 | 0.019±0.013 | 0.180 | 0.0002±0.0004 | 0.1061±0.2390 | 0.884 |
| **GLZLM_HGZE** | 110±94 | 121±59 | 0.525 | 3950±675 | 3777±1009 | 0.884 |
| **GLZLM_SZLGE** | 0.010±0.008 | 0.006±0.002 | 0.404 | 0.0002±0.0004 | 0.0096±0.0215 | 0.884 |
| **GLZLM_SZHGE** | 50±62 | 44±32 | 0.591 | 194±475 | 0.018±0.040 | 0.961 |
| **GLZLM_LZLGE** | 1790±4295 | 194±280 | 0.733 | 362±395 | 2249±2061 | **0.010** |
| **GLZLM_LZHGE** | 0.30E+6±0.33E+6 | 1.01E+6±1.21E+6 | 0.078 | 0.65E+10±0.70E+10 | 4.01E+10±3.68E+10 | **0.010** |
| **GLZLM_GLNU** | 5.4±2.3 | 11.3±6.8 | **0.020** | 1.00±0.00 | 1.06±0.21 | 0.808 |
| **GLZLM_ZLNU** | 14±18 | 22±25 | 0.301 | 1.12±0.29 | 1.00±0.00 | 0.591 |
| **GLZLM_ZP** | 0.08±0.09 | 0.05±0.04 | 0.733 | 0.0017±0.0018 | 0.0004±0.0005 | **0.048** |

*Abbreviations: FDG, fluorodeoxyglucose; PET, positron emission tomography; ADC, apparent diffusion coefficient; M0, no synchronous distant metastasis; M1, with synchronous distant metastasis; NA, not available; GLCM, Gray Level Cooccurrence Matrix; GLRLM, Gray level run length matrix; SRE, short-run emphasis; LRE = long-run emphasis; LGRE, low gray-level run emphasis; HGRE, High gray-level run emphasis; SRLGE, short-run low gray-level emphasis; SRHGE, Short-nun high gray-level emphasis; LRLGE, long-run low gray-level emphasis; LRHGE, Long-run high gray-level emphasis; GLNU, Gray-level non-uniformity; RLNU, Run length non-uniformity; RP, Run Percentage; GLZLM, Gray level zone length matrix; SZE, short-zone emphasis; LZE, Long-zone emphasis; LGZE, low gray-level zone emphasis; HGZE, High gray-level zone emphasis; SZLGE, short-zone low gray-level emphasis; SZHGE , short-zone high gray-level emphasis; LZLGE, long-zone low gray-level emphasis; LZHGE, Long-zone high gray-level emphasis; ZLNU, Zone length non-uniformity; and ZP, zone percentage.*
